# Developmental manifestations of polygenic risk for bipolar disorder from infancy to middle childhood

**DOI:** 10.1038/s41398-023-02522-2

**Published:** 2023-06-23

**Authors:** Ragna Bugge Askeland, Laurie J. Hannigan, Kevin S. O’Connell, Elizabeth C. Corfield, Oleksandr Frei, Anita Thapar, George Davey Smith, Ted Reichborn-Kjennerud, Ole A. Andreassen, Helga Ask, Alexandra Havdahl

**Affiliations:** 1 Department of Mental Disorders, Norwegian Institute of Public Health, 0473 Oslo, Norway; 2 Medical Research Council Integrative Epidemiology Unit, University of Bristol, Bristol, BS8 2BN UK; 3 Institute of Clinical Medicine, University of Oslo, Oslo, Norway; 4 Nic Waals Institute, Lovisenberg Diaconal Hospital, Spångbergveien 25, 0853 Oslo, Norway; 5 Center for Genetic Epidemiology and Mental Health, Norwegian Institute of Public Health, 0473 Oslo, Norway; 6 NORMENT Centre, Institute of Clinical Medicine, University of Oslo, Oslo, Norway; 7 NORMENT Centre, Division of Mental Health and Addiction, Oslo University Hospital, Oslo, Norway; 8 Division of Psychological Medicine and Clinical Neurosciences; Centre for Neuropsychiatric Genetics and Genomics; Wolfson Centre for Young People’s Mental Health, Cardiff University School of Medicine, Cardiff, Wales UK; 9 KGJ Centre for Neurodevelopment, Institute of Clinical Medicine, University of Oslo, Oslo, Norway; 10 PROMENTA Research Center, Department of Psychology, University of Oslo, 0373 Oslo, Norway

**Keywords:** Clinical genetics, Human behaviour, Bipolar disorder

## Abstract

Knowledge on how genetic risk for bipolar disorder manifests in developmental, emotional or behavioral traits during childhood is lacking. This issue is important to address to inform early detection and intervention efforts. We investigated whether polygenic risk for bipolar disorder is associated with developmental outcomes during early to middle childhood in the general population, and if associations differ between boys and girls. Our sample consisted of 28 001 children from the Norwegian Mother, Father and Child Cohort study, a prospective pregnancy cohort with available genotype and developmental data. Mothers reported on a range of developmental outcomes in their children at 6 and 18 months, 3, 5 and 8 years. Polygenic risk scores reflecting common variant liability to bipolar disorder were calculated. Linear regression models were used in a multi-group framework to investigate associations between polygenic risk score and developmental outcomes, using sex as a grouping variable. We found robust evidence for an association between polygenic risk scores for bipolar disorder and conduct difficulties (β = 0.041, CI = 0.020–0.062) and oppositional defiant difficulties (β = 0.032, CI = 0.014–0.051) at 8 years. Associations with most other outcomes were estimated within the region of practical equivalence to zero (equivalence range *D* = −0.1 to 0.1), with the exceptions of negative association for activity levels (β = −0.028, CI = −0.047– −0.010) at age 5 and benevolence (β = −0.025, CI = –0.043 to –0.008) at age 8, and positive association for motor difficulties (β = 0.025, CI = 0.008–0.043) at age 3, inattention (β = 0.021, CI = 0.003–0.041) and hyperactivity (β = 0.025, CI = 0.006–0.044) at age 8. Our results suggest that genetic risk for bipolar disorder manifests as disruptive behaviors like oppositional defiant and conduct difficulties in childhood in the general population.

## Introduction

Bipolar disorder is a complex psychiatric disorder typically diagnosed in young adulthood [1]. It affects mood, energy, activity, and concentration and is characterized by episodes of mania, hypomania and major depression. Bipolar disorder is ranked as one of the top causes of lost years of life and health in 15 to 44 year olds [2] and affects 2–3% of the population worldwide [3]. Despite the characteristic phenotype, bipolar disorder is often misdiagnosed, leading to inappropriate or delayed treatment which contributes to the high burden of morbidity and mortality [3, 4]. Observational studies suggest that early intervention might improve disease course and outcome, and delay to first treatment is associated with poorer outcomes [3, 5]. Earlier identification of warning signs, and improved intervention efforts would therefore be of great importance [6].

Bipolar disorder aggregates in families and twin studies report heritability of 60–80% [7, 8]. Molecular genetic studies have led to valuable insights about the genetic influence on bipolar disorder [9–11]. Genome wide association studies (GWAS) indicate that a substantial part of the genetic liability is conferred by common single nucleotide polymorphisms (SNPs) [12, 13]. In the most recent GWAS approximately 64 SNPs with genome-wide significant associations with bipolar disorder have been identified [10]. The variance explained by additive effects for bipolar disorder (SNP heritability) has been estimated to be 18.6% [10].

Polygenic risk scores (PRS) use data from large-scale GWAS and combine the effects of many common SNPs to capture the cumulative effect of risk alleles [14]. Risk alleles for bipolar disorders have been reported to overlap with other psychiatric diagnoses such as schizophrenia, major depressive disorders, autism spectrum disorder, anxiety disorders, and traits like intelligence [15–17].

Genetic risk for bipolar disorder might manifest in clinical and sub-clinical neurodevelopmental, emotional, or behavioral traits in the general population. The timing and nature of such associations could be informative for early identification efforts. High-risk prospective studies of the offspring of adults with bipolar disorder show elevated rates of behavioral disorders, Attention deficit/Hyperactivity disorder (ADHD), anxiety and depression as well as bipolar spectrum disorder in the offspring [18]. Bipolar PRS also are associated with risk of bipolar disorder in high-risk offspring [19]. These studies suggest that bipolar PRS may manifest early in development.

Prospective population-based cohorts, like the Norwegian Mother, Father and Child Cohort Study (MoBa), represent a unique framework for studying the associations between development, behavior and emotions, and genetic risk from an early age [20]. Only a few studies have used PRS to investigate associations between genetic risk for bipolar disorders and traits related to mental health in adulthood [21, 22], and as far as we know only two studies investigated associations in childhood [23, 24]. The knowledge on how PRS for bipolar disorder is associated with development, behavior and emotions during early childhood in the general population is still uncertain [24].

The potential for bipolar disorder PRS to be differently associated between sexes has been under-explored. The prevalence of bipolar disorder is similar in males and females, but females are more likely to experience rapid cycling and mixed states, and to have patterns of comorbidity that differ from males [1].

Access to the largest and most recent bipolar disorder GWAS provides increased statistical power. In the present study, we aimed to investigate whether, when, and how PRS for bipolar disorder is associated with development, behavior and emotions during early to middle childhood in the general population, in both males and females.

## Materials and methods

### Participants and measures

MoBa is a longitudinal prospective pregnancy cohort including approximately 114,500 children, their mothers, and fathers [25, 26]. Between 1999 and 2008 pregnant women were recruited to the study and gave written informed consent to participation in 41% of the pregnancies. Blood samples were collected from the children’s umbilical cord at birth [27]. The establishment of MoBa and initial data collection was based on a license from the Norwegian Data Protection Agency and approval from The Regional Committees for Medical and Health Research Ethics. MoBa is currently regulated by the Norwegian Health Registry Act. The current study was approved by The Regional Committees for Medical and Health Research Ethics (14140) and has undergone a Data Protection Impact Assessment.

Mother-reported questionnaire data was collected at different time points, during pregnancy and after birth. In our analyses we use data collected when the children were aged 6 and 18 months, 3, 5, and 8 years. These questionnaires includes several measures of developmental traits, as previously described elsewhere [28]. The current study is based on version 12 of the quality-assured data files released for research in January 2022.

The *phenotools*-package v0.2.7 (https://github.com/psychgen/phenotools) in R was used to prepare the outcome variables, and to ensure reproducibility. A mean score of the items was computed for each instrument, requiring at least half the items to be non-missing and multiplied by the number of items in the instrument to give a representative score on the scale of the instrument. Measures were reverse coded where necessary so that positive scores reflected higher scores on the measure.

*Social communication difficulties* were derived from the Ages and Stages Questionnaire (ASQ) scale at 6 months [29]. Both s*ocial communication difficulties* and *repetitive behavior* were assessed using items from the Modified Checklist for Autism in Toddlers [30, 31] at 18 months and items from the Social Communication Questionnaire [32] at 3 and 8 years. The mothers reported on a short version [33] of the Childhood Autism Spectrum Test [34] when the children were 5 years old.

*Inattention and hyperactivity/impulsivity* measures were derived using the Diagnostic and Statistical Manual of Mental Disorders (DSM)-oriented ADHD problems scale of the Child Behavior Check List (CBCL) [35] at 18 months and 3 years. At 5 years the revised Conner’s Parent Rating Scale [36] was used, and items from the Parent/Teacher Rating Scale for Disruptive Behavior Disorders (RS-DBD) [37] was used at 8 years.

*Disruptive behaviors* were assessed using items from the Aggressive behavior syndrome scale of the CBCL at age 18 months, 3 and 5 years for *aggression*, and by the RS-DBD at age 8 years, divided into measures of *oppositional defiant* and *conduct difficulties*.

*Language difficulties* were measured at 18 months, 3 and 5 years using the ASQ [29] and the Children’s Communication Checklist-2 [38] at 8 years. *Motor difficulties* were measured using the ASQ at 6 and 18 months, and the Children’s Development Inventory [39] at 5 years.

*Emotional difficulties* were assessed at 18 months, 3 and 5 years using the CBCL [40]. Anxiety and depressive signs were measured separately by the 5-item Screen for Child Anxiety Related Disorders [41] and the Short Mood and Feelings Questionnaire [42] at 8 years.

*Temperamental/personality* traits were measured at 6 months using the Infant Characteristics Questionnaire [43]. At 18 months, 3 and 5 years the Emotionality, Activity and Shyness Temperament Questionnaire [44] was used, and the Norwegian short form of the Hierarchical Personality Inventory for Children [45] was used at 8 years for the personality traits neuroticism, imagination, extraversion, conscientiousness and benevolence.

Details about each instrument is in the supplementary Text 1.

As a secondary set of outcomes to contextualize manifestations of bipolar disorder PRS in clinical terms, we extracted diagnostic outcomes from the Norwegian Patient Registry which contains information on diagnoses from the International Classification of Diseases, Tenth Revision (ICD-10) on in-and outpatients reported from all hospitals and specialized health care services in Norway from 2008-2019 [46]. We created the following diagnostic groups: *ADHD without conduct disorder* (ICD-10 code F900, F908 and F909, *n* = 1738), *Disruptive behavior disorders* (ICD-10 codes F91, F901 and F92, *n* = 348), *Autism spectrum diagnosis* (ICD-10 code F84, *n* = 332), *Affective disorders* (ICD-10 codes F31-F39, *n* = 164) and *Anxiety disorders* (ICD-10 codes F40, F41 and F93, *n* = 649).

### Genotyping and polygenic scores

The genotyping, imputation and quality control of the genetic data is described in the supplementary Text 2. Genotype data passing quality control filters was available for 28,001 unrelated children (47.9% female) of European genetic ancestry. Information on sex was retrieved from the Medical Birth Registry, which is a national health registry containing information about all births in Norway.

PRS for bipolar disorder were generated, using PRSice2 [47], for each child in our analytic sample, based on summary statistics from European samples from the most recent Psychiatric Genomic Consortium (PGC) GWAS for Bipolar Disorder (41,917 cases and 371,549 controls) [10]. Summary statistics from PGC was subject to standardized quality control as outlined in the original paper [10].

The PRS was adjusted for the covariates genotyping batch and population stratification. We used PRS built on 10 *p* value thresholds for inclusion of SNPs with progressively weaker associations with the disorder in the original GWAS. To avoid overlap between the PGC GWAS and our target sample, Norwegian participants were omitted from the PGC GWAS. A sub-sample from Norway consisting of 1883 adults with bipolar disorder and 47 237 controls were removed from the GWAS to make sure there could be no overlap in the discovery sample and our target sample.

To guard against inflated Type I error from overfitting, we performed a principal component analysis on the set of 10 PRS, using the *prcomp* function in R. The first principal component reweights the variants included to achieve maximum variation over all the 10 PRS thresholds used [48]. The first PRS principal component was used as the exposure variable in the regression models. All statistical analyses reflect associations between PRS principal components, but the abbreviation PRS will be used in the text.

### Statistical analyses

The *lavaan*-package v0.6-7 [49] was used to run multi-group linear regression models in R v3.6, estimating associations between the PRS and each of the outcome measures. Sex differences were investigated by including sex as a grouping variable. PRS and outcome measures were standardized to zero mean and unit variance prior to analyses.

To account for multiple testing, we corrected the critical p-value threshold corresponding to an alpha level of 5% for the number of effective tests run (Bonferroni correction). To determine the number of effective tests, we ran a principal component analysis on all 51 outcome measures [50]. The number of tests was defined as the number of principal components explaining 80% variance, which was 30. An alpha level of 5% is therefore reflected in a corrected *p* value of 0.0017 (0.05/30).

We used equivalence testing to examine whether estimated effects could be considered as equivalent to zero in practical terms. This can be done by setting bounds around the point null to create a region of practical equivalence to zero values for which are based on a selected *smallest effect size of interest* (SESOI). The equivalence testing procedure involves performing two one-sided tests to determine whether the effects at least as extreme as our SESOI can be rejected [51]. Objective SESOI-setting depends upon the intended application of the estimate (for example, as a tool for clinical stratification) and the strength and nature of the existing evidence for the effect in question. In the absence of an objectively agreed-upon SESOI, equivalence testing using a pre-specified SESOI can provide useful additional context when used in conjunction with null hypothesis significance testing (NHST). As such, we use a benchmark value of half a ‘small effect’ (i.e., Cohen’s d = 0.1) [52] as our SESOI as an agnostic starting point with the aim that, as this approach becomes more commonplace in the field, more informed SESOI will be possible.

The equivalence testing was also performed with an alpha level of 5%, after adjustment of multiple testing correction (as described above). However, it is important to note that because equivalence testing consists of two one-sided tests, both of which need to be significant to support a conclusion of practical equivalence to zero, this alpha level is preserved by assessing whether 90% (and not 95%) confidence intervals fall within the region of practical equivalence to zero. Therefore, the equivalence test results are presented with multiple testing-corrected 90% confidence intervals for straightforward interpretation in relation to the null region defined by the SESOI.

As a secondary set of analyses, to see if any patterns from the analyses of dimensional measures could be replicated for categorically defined diagnoses, we ran logistic regression models estimating the associations between the PRS and psychiatric diagnoses in childhood. These analyses are considered as secondary analyses due to power issues preventing sex stratification as per the primary analyses.

## Results

Descriptive statistics for the developmental outcomes are presented in Table 1 for males and females separately, including number of items and Cronbach’s alpha for each measure. Supplementary Fig. 1 shows the correlation between these outcomes.

**Table 1:** Descriptive statistics for measures of neurodevelopmental traits at all ages for children with genotype data.

Age	Developmental outcome	*N*	*N*	Mean (SD)	Mean (SD)	No of items in scale	cronbachs alpha
		boys	girls	boys	girls		
6 months	Emotionality	12702	11734	11.07 (1.62)	11.09 (1.64)	2	0.39
	Fussyness	12698	11731	9.50 (5.59)	9.08 (5.41)	7	0.68
	Motor difficulties	12708	11723	0.75 (1.24)	0.75 (1.22)	6	0.50
	Social communication	12705	11721	0.41 (0.75)	0.39 (0.74)	5	0.23
18 months	Sociability	10819	9899	8.88 (1.51)	8.94 (1.49)	2	0.32
	Shyness	10911	9949	2.99 (1.84)	3.24 (1.93)	3	0.64
	Activity	10913	9956	9.26 (1.93)	8.91 (1.92)	3	0.65
	Negative emotionality	10908	9949	5.22 (2.25)	5.15 (2.31)	3	0.64
	Emotional difficulties	10684	9829	1.25 (1.20)	1.31 (1.19)	5	0.41
	Aggression	10944	9982	7.20 (1.58)	6.97 (1.50)	5	0.43
	Inattention	10913	9948	3.17 (0.97)	3.07 (0.94)	2	0.30
	Hyperactive	10653	9787	3.52 (0.95)	3.42 (0.90)	2	0.49
	Motor difficulties	10922	9965	0.66 (1.28)	0.76 (1.31)	6	0.54
	Language difficulties	10901	9960	1.51 (1.60)	0.92 (0.32)	3	0.60
	Social communication	10661	9797	15.45 (0.87)	15.36 (0.77)	15	0.47
	Repetitive behavior	10662	9798	6.37 (0.63)	6.34 (0.61)	6	0.24
3 years	Sociability	8715	8049	7.94 (1.68)	8.21 (1.69)	3	0.52
	Shyness	8717	8044	3.55 (1.98)	3.69 (2.07)	3	0.67
	Activity	8718	8049	8.07 (2.11)	7.75 (2.08)	3	0.64
	Negative emotionality	8710	8047	5.26 (2.25)	5.47 (2.33)	3	0.65
	Emotional difficulties	8703	8022	2.06 (1.88)	2.25 (1.98)	9	0.55
	Aggression	8702	8022	7.49 (1.68)	7.23 (1.58)	5	0.56
	Inattention	8707	8029	3.22 (0.98)	3.16 (0.95)	2	0.48
	Hyperactive	8704	8025	6.29 (1.65)	6.22 (1.62)	4	0.64
	Motor difficulties	8684	8037	1.50 (1.43)	0.81 (1.10)	4	0.32
	Language difficulties	8721	8053	0.74 (1.24)	0.51 (0.88)	6	0.62
	Social communication	8715	8045	2.42 (1.87)	2.05 (1.56)	26	0.58
	Repetitive behavior	8702	8029	4.04 (2.56)	3.57 (2.41)	12	0.72
5 years	Sociability	6231	5924	9.13 (1.93)	9.36 (1.85)	3	0.71
	Shyness	6229	5926	3.23 (2.09)	3.25 (2.11)	3	0.70
	Activity	6230	5924	6.91 (2.21)	6.42 (2.05)	3	0.70
	Negative emotionality	6230	5923	4.22 (2.45)	4.28 (2.50)	3	0.75
	Emotional difficulties	6224	5925	1.91 (2.20)	2.04 (2.16)	11	0.66
	Aggression	6223	5926	6.55 (1.58)	6.35 (1.41)	5	0.57
	Inattention	6232	5926	12.74 (3.96)	11.85 (3.24)	9	0.86
	Hyperactive	6228	5920	4.29 (1.48)	4.00 (1.25)	3	0.56
	Motor difficulties	6225	5922	10.84 (1.63)	11.50 (1.05)	12	0.71
	Language difficulties	6233	5942	3.13 (2.39)	2.63 (2.29)	6	0.59
	Social communication	2509	2337	11.76 (1.03)	11.60 (0.91)	11	0.44
	Repetitive behavior	2507	2334	5.48 (0.72)	5.37 (0.63)	5	0.30
8 years	Neuroticism	6514	6074	7.71 (4.69)	7.59 (4.43)	6	0.57
	Imagination	6521	6081	17.43 (3.46)	18.34 (3.29)	6	0.69
	Extraversion	6518	6074	15.76 (3.87)	16.94 (3.59)	6	0.63
	Concentiousness	6522	6082	15.54 (3.66)	16.32 (3.63)	6	0.75
	Benevolence	6523	6081	15.40 (3.86)	15.57 (3.74)	6	0.22
	Oppositional defiant difficulties	6523	6082	3.70 (3.37)	3.26 (3.01)	8	0.84
	Conduct difficulties	6533	6089	1.09 (1.85)	0.50 (1.13)	8	0.70
	Depression	6517	6076	1.92 (2.53)	1.79 (2.39)	13	0.79
	Anxiety	6529	6086	0.92 (1.15)	1.09 (1.18)	5	0.47
	Inattention	6529	6080	5.79 (4.63)	4.31 (3.80)	9	0.87
	Hyperactive	6528	6082	4.26 (4.50)	3.09 (3.67)	9	0.86
	Language difficulties	6515	6070	5.73 (5.14)	4.75 (4.48)	16	0.82
	Social communication	6505	6056	2.87 (2.53)	2.30 (2.26)	26	0.70
	Repetitive behavior	6535	6085	0.75 (1.27)	0.54 (1.00)	12	0.61

Note: The difference in data availability for the repetitive behavior and social communication variable at 5 years compared to other measures in Table 1 is due to the version of the 5-year questionnaire containing those measures being sent only to a subset of MoBa participants.

### PRS for Bipolar Disorder and developmental outcomes

**Fig. 1:**
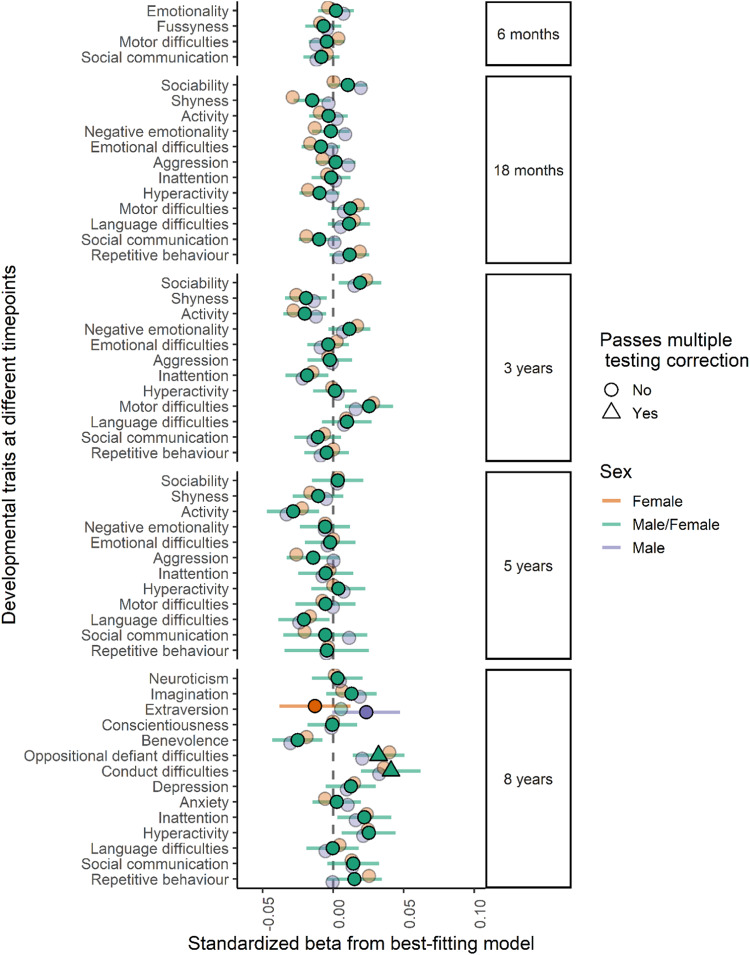
Bipolar disorder PRS and developmental outcomes. PRS for Bipolar disorder and measures of repetitive behavior, social communication difficulties, language and motor difficulties, hyperactivity, inattention, anxiety, depression, emotional difficulties, fussiness, and trait measures of emotionality, activity, shyness and sociability and personality trait measures benevolence, conscientiousness, extraversion, imagination, and neuroticism. Estimates from linear regression models with sex as a grouping variable in a multi group framework. The darker fill intensity indicate which model (sex difference or no sex difference) provided a better fit to the PRS. Estimates from the better-fitting (sex difference or no sex difference) model also have 95% confidence interval bars, whilst those from the poorer-fitting model are presented only as point estimates for reference. Results presented in a triangle means they passed multiple testing correction with a *p* value < 0.0017, corresponding to an alpha of 5%.

The associations between bipolar disorder PRS and developmental outcomes at different time points and in males and females are shown in Fig. 1. Only two outcomes passed multiple testing correction, and we found no robust evidence of sex differences. There was robust evidence for a sex invariant association with conduct difficulties (β = 0.041, CI = 0.020–0.062, *P* = 0.0001) and oppositional defiant difficulties (β = 0.032, CI = 0.014–0.051, *P* = 0.0006).

**Fig. 2:**
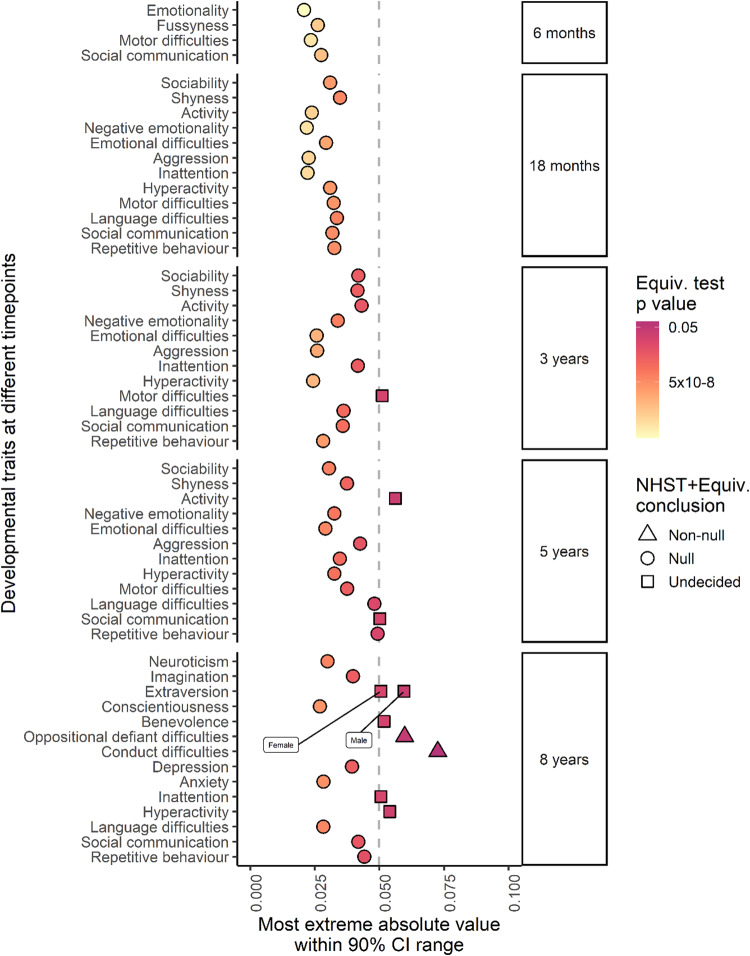
Equivalence test results for all developmental outcomes. This figure shows the categorizations in the context of the most extreme absolute value within the multiple testing-corrected 90% confidence interval range for each measure, in relation to the SESOI. Where these values are less than the SESOI, the equivalence test result estimated within the region of practical equivalence to zero. Bipolar disorder PRS and developmental outcomes of repetitive behavior, social communication difficulties, language and motor difficulties, hyperactivity, inattention, anxiety, depression, emotional difficulties, fussiness, emotionality, activity, shyness, sociability, benevolence, conscientiousness, extraversion, imagination, and neuroticism. The null hypothesis in the table refers to a composite null hypothesis of the NHST plus equivalence test. Results presented in a triangle means the composite null test could be rejected. Results presented as circles means they could not be rejected, and results presented as squares means it remains undecided.

The results from the equivalence testing on the associations between bipolar disorder PRS and developmental outcomes are shown in Fig. 2. Effects are categorized as either (a) null effects (estimated entirely within the region of practical equivalence to zero); (b) non-null effects (estimated at least partly outside the region of practical equivalence to zero *and* entirely distinct from the point null); or (c) effects about which we must remain undecided (estimated at least partly outside the region of practical equivalence to zero but not distinct from the point null). See supplementary information for equivalence testing results with the SESOI bounds and multiple testing-adjusted 90% confidence intervals (Supplementary Fig. 2). Figure 2 shows the categorisations in the context of the most extreme absolute value within the multiple testing-corrected 90% confidence interval range for each measure, in relation to the SESOI. The majority of effect estimates could be declared as null on the basis of the equivalence test results. We did not identify any sex differences in the equivalence testing. Estimates for oppositional defiant and conduct difficulties at age 8 were non-null. In the case of motor difficulties at age 3, activity levels at age 5, and benevolence, inattention, and hyperactivity at age 8 the data are inconclusive for these outcomes. Results were also inconclusive for social communications difficulties at 5 years and extraversion at 8 years, but it is worth noting that effects in these domains were estimated substantially less precisely due to, respectively, low sample size and sex differentiation. This means that although practical equivalence to zero was not supported for these outcomes, their point estimates were not particularly extreme.

**Fig. 3:**
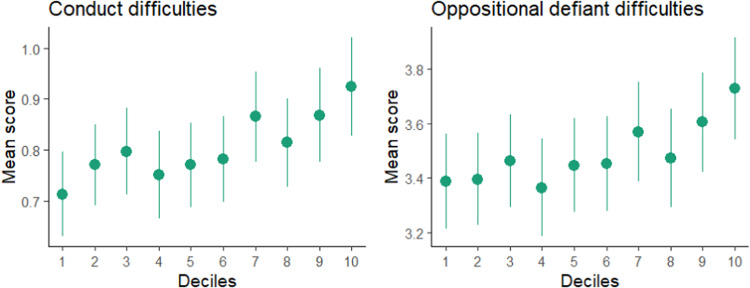
Decile plot for PRS for bipolar disorder and conduct difficulties and oppositional defiant difficulties. Decile plots with confidence intervals of the mean at each decile. The plots demonstrate the increased risk among individuals from the bottom to the top percentiles of Bipolar disorder PRS, relative to individuals in the middle of the distribution.

Figure 3 shows how the bipolar disorder PRS associates with conduct and oppositional defiant difficulties as the burden of risk variants increases. The plots demonstrate the decreased risk among individuals in the top and bottom deciles of PRS, relative to individuals with PRS in the middle of the distribution. For conduct difficulties, our results show increased risk in the top 90% percentile compared to the bottom 10% with non-overlapping confidence intervals, but not different from the individuals in the middle of the distribution. The confidence intervals overlap between all percentiles for oppositional defiant difficulties, although individuals in the top decile had higher mean score than those in the bottom decile.

### PRS for bipolar disorder and diagnostic outcomes

**Fig. 4:**
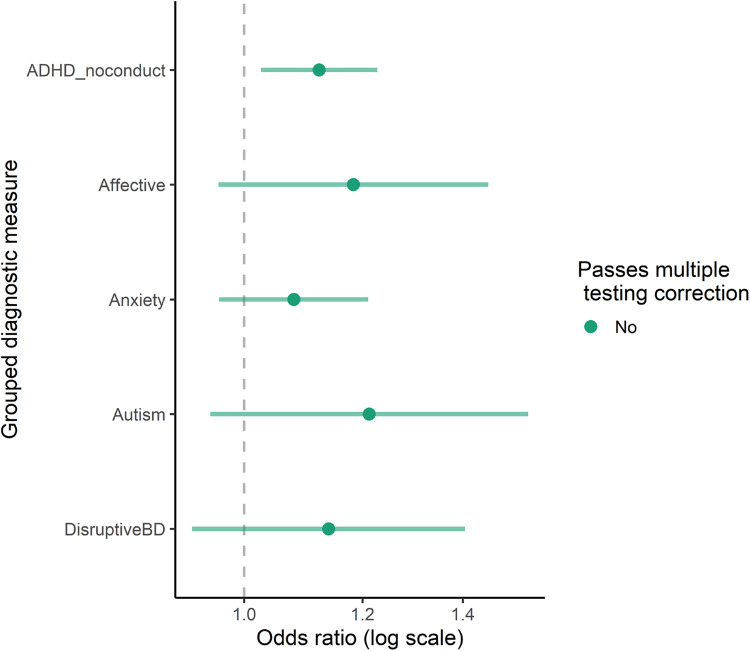
Bipolar disorder PRS and diagnostic groups. PRS for Bipolar disorder and grouped diagnostic measures. ADHD_noconduct; ADHD without conduct disorder (combined F900, F908, F909), Affective; affective disorder (combined F31-F39), Anxiety; anxiety disorders (combined F40, F41, F93), autism; (F84), DisruptiveBD; Disruptive behavior disorder (combined F91, F901, F92). Estimates from logistic regression model. Estimates shown with 95% confidence interval bars.

The associations between bipolar disorder PRS and grouped diagnostic measures are shown in Fig. 4. None of the outcomes passed multiple testing correction. However, the results from the equivalence testing indicated effects larger than the SESOI could not be ruled out (Supplementary Table 1) for any grouped diagnostic measures, suggesting that more data are needed to draw conclusive inferences about the presence or absence of effects for these outcomes.

As a *post hoc* analysis we investigated if the criteria listed in DSM-5 for oppositional defiant difficulties (*irritable mood, defiant behavior and vindictiveness*) and conduct difficulties (*aggression, deceitfulness, destruction, violation of rules*), measured dimensionally in MoBa, were associated with our bipolar disorder PRS, these results are presented in Supplementary Fig. 3. We identified an association for bipolar disorder PRS and defiant behavior, vindictiveness and aggression.

## Discussion

We investigated the associations between bipolar disorder PRS and a range of developmental outcomes from infancy to middle childhood. Using results from the largest and most recent GWAS on bipolar disorder we calculated PRS in the largest genotyped population-based pregnancy cohort to date. We found robust evidence for an association with oppositional defiant and conduct difficulties at 8 years. These associations were large enough to be categorized as non-null, falling outside a pre-specified region of practical equivalence to zero based on a SESOI of 0.1 SDs. We remain undecided on whether bipolar disorder PRS is associated with motor difficulties at age 3, activity levels at age 5, and benevolence, inattention, and hyperactivity at age 8, and the grouped diagnostic measures. Most other observed associations were equivalent to zero.

Our main finding is that bipolar disorder PRS manifests in childhood oppositional defiant and conduct difficulties. This is in line with findings from family studies showing that offspring of parents with bipolar disorder are at an increased risk of developing oppositional defiant or conduct disorder, compared to offspring of parents without bipolar disorder [18, 53, 54]. Our observation is also supported by clinical studies reporting lifetime comorbidity of bipolar and conduct disorder [55, 56]. They suggest that conduct disorder might be predictive of future bipolar disorder or account for the failure of early detection of bipolar disorder.

We could not find any studies investigating the association between bipolar disorder PRS and oppositional defiant or conduct difficulties specifically. We explored if listed criteria from DSM-5, measured dimensionally in MoBa, drives the association observed in our sample. The results showed associations with *defiant behavior* and *vindictiveness*, but we were unable to identify an association with *irritable mood*. According to DSM-5 [1] it is not unusual to show the behavioral features without irritable mood in children with oppositional defiant difficulties.

Categorical definitions of psychiatric disorders may not be optimal for investigating associations of genetic risk [57]. The equivalence testing indicated that effects larger than the SESOI could not be ruled out. There were few individuals with a diagnosis in our sample, which most likely explains why we were unable to find any robust associations.

Like other big cohort studies, we identified only a few robust associations with childhood development. In the Avon Longitudinal Study of Parents and Children (ALSPAC) including 6 105 children, aged 7–9 years, PRS based on a smaller discovery sample for bipolar disorder had a robust association with ADHD, while no strong evidence was found for association with emotional or behavioral difficulties [24]. In a meta-analysis including 42,998 individuals aged 6–17 years, no strong evidence for associations between PRS for bipolar disorder and any measured childhood emotional, behavioral or neurodevelopmental trait was identified [23]. Bipolar disorder has been suggested to be a neurodevelopmental disorder by some [58, 59], and according to a Dutch twin study [60] one would then expect the first signs of the illness to manifest early in development and before first manic or depressive episode. In the Pittsburgh Bipolar Offspring study, 75.6% of children of parents with bipolar disorder who themselves also developed bipolar disorder had onset prior to age 12 [18]. Our PRS associations with disruptive behaviors were only robust after age 8 years, suggesting a certain degree of maturation is required before the genetic vulnerability is expressed in observable behavior. Our analyses should be followed up when data from the 14-year questionnaire have been released from MoBa. It will be important to examine if the PRS associations are then more robust and broader, and if any sex differences are detected.

Our study is not without limitations. Some limitations are unique to analyses using PRS based on GWAS. For example the GWAS sample size will affect how many SNPs have been identified as risk SNPs, and the accuracy of individual predictions rely on the size of the GWAS [61]. So far GWAS yield small effect sizes, and ascertainment methods both in the GWAS sample and the target sample will affect the accuracy of PRS [10]. GWAS do not capture rare genetic variants, so only associations with common variants are investigated. Some limitations are specific to using data from MoBa. It is important to note that, the available measures at various ages are not identical across timepoints, they are not analyzed as developmental trajectories, but are specific for given time points for each measure. We used of one of the largest prospective population-based pregnancy cohorts worldwide, but the current subsample may not be adequately powered to identify small effects in some of the measured domains. MoBa is subject to attrition, just like all longitudinal studies [62]. Previous studies have shown that predictors of attrition include presence of behavioral difficulties in the study child [63]. Selective attrition could lead to bias in our estimates; likely in the form of an underestimation of associations between the PRS and the developmental traits. Future studies should investigate this, but it might be that power will be a limitation until sample sizes increase or the predictive power of PRS is substantially enhanced. PRS combined with cognitive performance tests, cortical thickness measures and gray matter density maps, might have increased classification performance, but these findings need to be investigated further [64].

In conclusion, our results suggest that genetic risk for bipolar disorder, as indexed by PRS, might manifest as disruptive behaviors in childhood in the general population. In the case of motor difficulties, activity levels, social communication difficulties, benevolence, inattention, extraversion, and hyperactivity the data were inconclusive. It will be important to examine if the PRS associations are more robust and if any sex differences are detected with a bigger sample and measures available at an older age.

## Supplementary information

Supplementary material

## References

[1] APA. *Diagnostic and statistical manual of mental disorders (DSM-5®)*, vol. Fifth edition. American Psychiatric Association; 2013.

[2] Whiteford HA, Degenhardt L, Rehm J, Baxter AJ, Ferrari AJ, Erskine HE (2013). Global burden of disease attributable to mental and substance use disorders: findings from the Global Burden of Disease Study 2010. Lancet.

[3] Bauer M, Andreassen OA, Geddes JR, Kessing LV, Lewitzka U, Schulze TG (2018). Areas of uncertainties and unmet needs in bipolar disorders: clinical and research perspectives. Lancet Psychiatry.

[4] Ferrari AJ, Stockings E, Khoo JP, Erskine HE, Degenhardt L, Vos T (2016). The prevalence and burden of bipolar disorder: findings from the Global Burden of Disease Study 2013. Bipolar Disord.

[5] Post RM, Leverich GS, Kupka RW, Keck PE, McElroy SL, Altshuler LL (2010). Early-onset bipolar disorder and treatment delay are risk factors for poor outcome in adulthood. J Clin Psychiatry.

[6] Sullivan PF, Agrawal A, Bulik CM, Andreassen OA, Børglum AD, Breen G (2018). Psychiatric genomics: an update and an agenda. Am J Psychiatry.

[7] Fabbri C. The role of genetics in bipolar disorder. Bipolar disorder: from neuroscience to treatment. 2021:41–60. 10.1007/7854_2020_153 32767004

[8] Johansson V, Kuja-Halkola R, Cannon TD, Hultman CM, Hedman AM (2019). A population-based heritability estimate of bipolar disorder–In a Swedish twin sample. Psychiatry Res.

[9] Craddock N, Sklar P (2013). Genetics of bipolar disorder. Lancet.

[10] Mullins N, Forstner AJ, O’Connell KS, Coombes B, Coleman JR, Qiao Z (2021). Genome-wide association study of more than 40,000 bipolar disorder cases provides new insights into the underlying biology. Nat Genet.

[11] Charney A, Ruderfer D, Stahl E, Moran J, Chambert K, Belliveau R (2017). Evidence for genetic heterogeneity between clinical subtypes of bipolar disorder. Transl Psychiatry.

[12] Sullivan PF, Daly MJ, O’Donovan M (2012). Genetic architectures of psychiatric disorders: the emerging picture and its implications. Nat Rev Genet.

[13] Grove J, Ripke S, Als TD, Mattheisen M, Walters RK, Won H (2019). Identification of common genetic risk variants for autism spectrum disorder. Nat Genet.

[14] Wray NR, Lee SH, Mehta D, Vinkhuyzen AA, Dudbridge F, Middeldorp CM (2014). Research review: polygenic methods and their application to psychiatric traits. J child Psychol Psychiatry.

[15] McIntyre RS, Berk M, Brietzke E, Goldstein BI, López-Jaramillo C, Kessing LV (2020). Bipolar disorders. Lancet.

[16] Cross-Disorder Group of the Psychiatric Genomics Consortium. Identification of risk loci with shared effects on five major psychiatric disorders: a genome-wide analysis. Lancet. 2013;381:1371–9. 10.1016/S0140-6736(12)62129-1 PMC3714010 23453885

[17] Cross-Disorder Group of the Psychiatric Genomics Consortium. Genetic relationship between five psychiatric disorders estimated from genome-wide SNPs. Nat Genet. 2013;45:984. 10.1038/ng.2711 PMC3800159 23933821

[18] Birmaher B, Axelson D, Monk K, Kalas C, Goldstein B, Hickey MB (2009). Lifetime psychiatric disorders in school-aged offspring of parents with bipolar disorder: the Pittsburgh Bipolar Offspring study. Arch Gen Psychiatry.

[19] Birmaher B, Hafeman D, Merranko J, Zwicker A, Goldstein B, Goldstein T (2022). Role of polygenic risk score in the familial transmission of bipolar disorder in youth. JAMA Psychiatry.

[20] Robinson JP, Shaver PR, Wrightsman LS. *Measures of personality and social psychological attitudes: Measures of social psychological attitudes*, Academic Press 2013 vol. 1.

[21] Schulze TG, Akula N, Breuer R, Steele J, Nalls MA, Singleton AB (2014). Molecular genetic overlap in bipolar disorder, schizophrenia, and major depressive disorder. World J Biol Psychiatry.

[22] Power RA, Steinberg S, Bjornsdottir G, Rietveld CA, Abdellaoui A, Nivard MM (2015). Polygenic risk scores for schizophrenia and bipolar disorder predict creativity. Nat Neurosci.

[23] Akingbuwa WA, Hammerschlag AR, Jami ES, Allegrini AG, Karhunen V, Sallis H (2020). Genetic associations between childhood psychopathology and adult depression and associated traits in 42 998 individuals: a meta-analysis. JAMA Psychiatry.

[24] Mistry S, Escott-Price V, Florio AD, Smith DJ, Zammit S (2019). Genetic risk for bipolar disorder and psychopathology from childhood to early adulthood. J Affect Disord.

[25] Magnus P, Birke C, Vejrup K, Haugan A, Alsaker E, Daltveit AK (2016). Cohort profile update: the Norwegian mother and child cohort study (MoBa). Int J Epidemiol.

[26] Magnus P, Irgens LM, Haug K, Nystad W, Skjærven R, Stoltenberg C (2006). Cohort profile: the Norwegian mother and child cohort study (MoBa). Int J Epidemiol.

[27] Paltiel L, Anita H, Skjerden T, Harbak K, Bækken S, Kristin SN, et al. The biobank of the Norwegian Mother and Child Cohort Study–present status. Norsk epidemiologi. 2014:24.1–2.

[28] Askeland RB, Hannigan LJ, Ask H, Ayorech Z, Tesli M, Corfield E, et al. Early manifestations of genetic risk for neurodevelopmental disorders. J Child Psychol Psychiatry 2022:63.7:810–819. 10.1111/jcpp.13528 PMC7616991 34605010

[29] Richter J, Janson H (2007). A validation study of the norwegian version of the ages and stages questionnaires. Acta Paediatr.

[30] Baron-Cohen S, Allen J, Gillberg C (1992). Can autism be detected at 18 months? The needle, the haystack and the CHAT. Br J Psychiatry.

[31] Robins DL, Fein D, Barton ML, Green JA (2001). The Modified Checklist for Autism in Toddlers: an initial study investigating the early detection of autism and pervasive developmental disorders. J Autism Dev Disord.

[32] Rutter M, Bailey A, Lord C. SCQ: Social Communication Questionnaire (Western Psychological Services, Los Angeles, 2003).

[33] Ronald A, Happé F, Plomin R (2008). A twin study investigating the genetic and environmental aetiologies of parent, teacher and child ratings of autistic-like traits and their overlap. Eur Child Adolesc Psychiatry.

[34] Scott FJ, Baron-Cohen S, Bolton P, Brayne C (2002). The CAST (Childhood Asperger Syndrome Test) Preliminary development of a UK screen for mainstream primary-school-age children. Autism.

[35] Achenbach TM, Dumenci L, Rescorla LA. Ratings of relations between DSM-IV diagnostic categories and items of the CBCL/6-18, TRF, and YSR. Burlington, VT: University of Vermont; 2001, 1–9.

[36] Kumar G, Steer RA (2003). Factorial validity of the Conners’ Parent Rating Scale-revised: short form with psychiatric outpatients. J Personal Assess.

[37] Silva RR, Alpert M, Pouget E, Silva V, Trosper S, Reyes K (2005). A rating scale for disruptive behavior disorders, based on the DSM-IV item pool. Psychiatr Q.

[38] Bishop DV. *The children’s communication checklist: CCC-2*. Harcourt Assessment 2003.

[39] Ireton H. Child development inventory. Behavior Science Systems Minneapolis, MN; 1992.

[40] Achenbach TM, Howell CT, Aoki MF, Rauh VA (1993). Nine-year outcome of the Vermont intervention program for low birth weight infants. Pediatrics.

[41] Birmaher B, Brent DA, Chiappetta L, Bridge J, Monga S, Baugher M (1999). Psychometric properties of the Screen for Child Anxiety Related Emotional Disorders (SCARED): a replication study. J Am Acad child Adolesc psychiatry.

[42] Messer SC, Angold A, Costello EJ, Loeber R, Van Kammen W, Stouthamer-Loeber M (1995). Development of a short questionnaire for use in epidemiological studies of depression in children and adolescents: Factor composition and structure across development. Int J methods Psychiatr Res.

[43] Bates JE, Freeland CAB, Lounsbury ML. Measurement of infant difficultness. Child Develop. 1979:794–803. 498854

[44] Buss AH, Plomin R. *Temperament: Early developing personality traits*. Hillsdale, NJ: Lawrence Erlbaum Associates; 1984.

[45] Vollrath ME, Hampson SE, Torgersen S (2016). Constructing a short form of the hierarchical personality inventory for children (HiPIC): the HiPIC‐30. Personal Ment health.

[46] Bakken IJ, Ariansen AM, Knudsen GP, Johansen KI, Vollset SE (2020). The Norwegian Patient Registry and the Norwegian Registry for Primary Health Care: Research potential of two nationwide health-care registries. Scand J Public Health.

[47] Choi SW, O’Reilly PF (2019). PRSice-2: Polygenic Risk Score software for biobank-scale data. Gigascience.

[48] Coombes BJ, Ploner A, Bergen SE, Biernacka JM (2020). A principal component approach to improve association testing with polygenic risk scores. Genet Epidemiol.

[49] Rosseel Y (2012). Lavaan: An R package for structural equation modeling and more. Version 0.5–12 (BETA). J Stat Softw.

[50] Leppert B, Havdahl A, Riglin L, Jones HJ, Zheng J, Smith GD (2019). Association of maternal neurodevelopmental risk alleles with early-life exposures. JAMA Psychiatry.

[51] Lakens D, Scheel AM, Isager PM (2018). Equivalence testing for psychological research: a tutorial. Adv Methods Pract Psychol Sci.

[52] Maxwell SE, Lau MY, Howard GS (2015). Is psychology suffering from a replication crisis? What does “failure to replicate” really mean?. Am Psychol.

[53] Birmaher B, Axelson D, Goldstein B, Monk K, Kalas C, Obreja M (2010). Psychiatric disorders in preschool offspring of parents with bipolar disorder: the Pittsburgh Bipolar Offspring Study (BIOS). Am J Psychiatry.

[54] Singh MK, DelBello MP, Stanford KE, Soutullo C, McDonough-Ryan P, McElroy SL (2007). Psychopathology in children of bipolar parents. J Affect Disord.

[55] Kutcher S, Marton P, Korenblum M (1989). Relationship between psychiatric illness and conduct disorder in adolescents. Can J Psychiatry.

[56] Kovacs M, Pollock M (1995). Bipolar disorder and comorbid conduct disorder in childhood and adolescence. J Am Acad Child Adolesc Psychiatry.

[57] Taylor MJ, Martin J, Lu Y, Brikell I, Lundström S, Larsson H (2019). Association of genetic risk factors for psychiatric disorders and traits of these disorders in a Swedish population twin sample. JAMA Psychiatry.

[58] Blumberg HP, Kaufman J, Martin A, Charney DS, Krystal JH, Peterson BS (2004). Significance of adolescent neurodevelopment for the neural circuitry of bipolar disorder. Ann NY Acad Sci.

[59] van Os J, Jones P, Lewis G, Wadsworth M, Murray R (1997). Developmental precursors of affective illness in a general population birth cohort. Arch Gen Psychiatry.

[60] Vonk R, Van Der Schot A, Van Baal G, Van Oel C, Nolen W, Kahn R (2012). Premorbid school performance in twins concordant and discordant for bipolar disorder. J Affect Disord.

[61] Dudbridge F (2013). Power and predictive accuracy of polygenic risk scores. PLoS Genet.

[62] Nilsen RM, Surén P, Gunnes N, Alsaker ER, Bresnahan M, Hirtz D (2013). Analysis of self‐selection bias in a population‐based cohort study of autism spectrum disorders. Paediatr Perinat Epidemiol.

[63] Wolke D, Waylen A, Samara M, Steer C, Goodman R, Ford T (2009). Selective drop-out in longitudinal studies and non-biased prediction of behaviour disorders. Br J Psychiatry.

[64] Doan NT, Kaufmann T, Bettella F, Jørgensen KN, Brandt CL, Moberget T (2017). Distinct multivariate brain morphological patterns and their added predictive value with cognitive and polygenic risk scores in mental disorders. NeuroImage: Clin.

