# Supplementary material for: Developmental manifestations of polygenic risk for bipolar disorder from infancy to middle childhood
Source: Transl Psychiatry. 2023 Jun 23;13:222. doi: 10.1038/s41398-023-02522-2 (PMC10290060; doi:10.1038/s41398-023-02522-2)
Supplement: Supplementary file 1 — Supplementary material [file 41398_2023_2522_MOESM1_ESM.pdf]

Supplementary Information content

Table of contents

Supplementary Text 1. Supplementary information about the scales used 2

Supplementary Text 2. Supplementary information about the genotyped data 6

Supplementary Figure 1. Correlation matrix of the dimensional measures 9

Supplementary Figure 2. Equivalence test results with SESOI bounds and widened confidence intervals 10

Supplementary Figure 3. PRS for Bipolar disorder and association with DSM-5 criteria for oppositional defiant difficulties and conduct difficulties 12

Supplementary Table 1. Equivalence test results for diagnostic measures 13

Supplementary references 14

Supplementary Text 1. Supplementary information about the scales used

For full scales used in MoBa see: https://mobawiki.fhi.no/mobawiki/index.php/Questionnaires.

Age at measure	Social communication and repetitive behavior measures	Inattention and hyperactivity measures	Disruptive behavior measures	Language difficulties	Motor difficulties	Emotional difficulties	Temperament and personality measures
6m	ASQ				ASQ		ICQ
18m	M-CHAT	CBCL	CBCL	ASQ	ASQ	CBCL	EAS
3y	SCQ	CBCL	CBCL	ASQ	ASQ	CBCL	EAS
5y	CAST	Connors (5y)	CBCL	ASQ	CDI (5y)	CBCL	EAS
8y	SCQ	RS-DBD (8y)	RS-DBD (8y)	CCC-2 (8y)		SMFQ, SCARED	NHiPIC

Ages and stages questionnaire (ASQ): In this study used for measures on social communication difficulties, language and motor difficulties at 6 months, 18 months, 3 years and 5 years. The ASQ is a series of screening questionnaires for children and has been found to be an effective screening tool for developmental difficulties[1, 2]. In MoBa it includes questions on motor difficulties like: "Can your child walk well and seldom fall?", language difficulties: "Does your child say eight or more words in addition to mama and dada?" and social communication difficulties: like "Does your child babble and make sounds when he/she is lying on his/her own?".

The Modified Checklist for Autism in Toddlers (M-CHAT): In this study used for measuring social communication difficulties and repetitive behaviour at 18 months. The M-CHAT[3] is a 23 yes/no checklist for parents to report on their child. It includes questions on social communication difficulties like "Does your child take an interest in other children?" and repetitive behaviour like "Does your child make unusual finger movements near his/her face?"

Social Communication Questionnaire (SCQ): Used for measuring social communication difficulties and repetitive behaviour at 3 and 8 years. The SCQ is a parent-reported questionnaire used in screening for autism[4], with questions about repetitive behaviour: "Does your child ever use odd phrases or say the same thing over and over again in almost exactly the same way? (either phrases that the child hears other people use or ones that he/she makes up" or "Does your child ever say the same thing

over and over in exactly the same way or insist that you say the same thing over and over again?”. Questions about social communication difficulties like “Does your child smile back if someone smiles at him/her?” and “Does your child’s facial expression usually seem appropriate to the particular situation, as far as you can tell?”.

Childhood Autism Spectrum Test (CAST; formerly named Childhood Asperger Syndrome Test): Used for measuring social communication difficulties and repetitive behaviour at 5 years[5]. Questions like “Does s/he find it easy to interact with other children?” is an example of social communication, and for repetitive behaviour there are questions like “Does s/he like to do things over and over again, in the same way all the time?”

The Children’s Communication Checklist-2 (CCC-2): Used for measuring language difficulties at 8 years. The CCC-2 was designed to measure children’s’ communication skills from 4 to 16 years[6]. It includes questions like: “It is hard to make sense of what s/he is saying, even though the words are clearly spoken” and “Does your child use four- and five- word sentences? For example, does your child say, “I want the car”?”

Child Behavior Check List (CBCL): The full CBCL scale is designed to identify children’s’ problem behaviour[7]. In this study we used the Diagnostic and Statistical Manual of Mental Disorders (DSM)-oriented Attention deficit/Hyperactivity disorder (ADHD) problems subscale divided into hyperactivity and inattention at 18 months and 3 years. Example inattention: “Can’t concentrate, pay attention for long”, example hyperactivity: “can’t sit still, restless or hyperactive”. At 18 months, 3 years and 5 years we used the aggression subscale with statements like “Gets into many fights” and “Hits others”. The CBCL also has an internalizing behavior measure, used for the Emotional difficulties measure in the study. Example of statements; “Clings to adults or too dependent”, “Disturbed by any change in routine”. These measures were available at 18 months, 3 years and 5 years.

Conners' Parent Rating Scale (CPRS-R): The CPRS-R[8] is used for measuring hyperactivity and inattention at 5 years with statements like "short attention span" and "fidgets with hands or feet, squirms in seat".

Parent/Teacher Rating Scale for Disruptive Behavior Disorders (RS-DBD): Used for measuring hyperactivity, inattention, oppositional deviance and conduct disorder at 8 years. The RS-DBD[9] consists of 41 DSM-IV items; with 9 items related to inattention, 9 items for hyperactivity, 8 items related to oppositional defiant difficulties, and 8 items to conduct difficulties. Inattention: "Has difficulty sustaining attention in tasks or play activities" and hyperactivity: "Runs about or climbs excessively in situations in which it is inappropriate". Oppositional defiant difficulties: "Actively defies or refuses to comply with adults' requests or rules", and conduct difficulties: "Has deliberately destroyed other's property.

Child Development Inventory (CDI): Used for measuring motor difficulties at 5 years. The CDI[10] was designed as a parent-reported questionnaire to measure child development, it has a subscale on motor development, and 10 of these questions are included in MoBa. An example of motor difficulties: "Puts together a puzzle with nine or more pieces".

Screen for Child Anxiety Related Disorders (SCARED): The SCARED[11] is a questionnaire developed to measure DSM-defined anxiety symptom. It contains 41 items which can be allocated to five separate anxiety subscales. The 5-item short version, as used in the MoBa, was developed by Birmaher, Brent [12] in 1999. It includes questions like: "My child is scared to go to school".

Short Mood and Feelings Questionnaire (SMFQ): Was developed to measure depression based on the DSM-III criteria[13]. A 13-item short form was developed, based on the discriminating ability between the depressed and non-depressed[14] and is the one used in MoBa at 8 years. It includes questions like: "Thought nobody really loved him/her" and "Felt s/he did everything wrong".

Infant Characteristics Questionnaire (ICQ): Used to measure temperament at 6 months. The ICQ[15] was designed as a parent-reported questionnaire comprised of 24 items. In MoBa the questions

about fuzzy/difficult subscale has been used. An example from the questionnaire: “The child is easily upset”.

The Emotionality, Activity and Shyness Temperament Questionnaire (EAS) was used at 18 months, 3 and 5 years to measure 4 temperament dimensions: shyness (fear), emotionality (irritability/anger), sociability (positive affect/including approach) and activity[16]. Three questions from each dimension is used in MoBa and constitute the short form of the questionnaire. Examples of questions about shyness: “Your child takes a long time to warm up to strangers”, emotionality: “Your child reacts intensely when upset”, activity: “Your child is always on the go” and sociability: “Your child likes to be with people”.

Short Norwegian Hierarchical Inventory for the Assessment of Personality in Children (NHiPIC): was used at 8 years in MoBa to measure the Big Five personality trait factors; Extraversion, Benevolence, Neuroticism, Conscientiousness, and Imagination[17]. In MoBa the 30-item short form was used, also referred to as NHiPIC-30. Examples of questions used in NHiPIC: “Talks to people easily” (extraversion), and “Has confidence in own abilities” (neuroticism).

Supplementary Text 2. Supplementary information about the genotyped data

Quality control (QC) of the genotyped individuals in MoBa is ongoing. Approximately 238,000 individuals have been genotyped from the Norwegian Mother, Father and Child cohort, and about 83,500 of them were children. At the time of analyses, approximately 98,000 individuals were genotyped and QC'ed. Genotypes were called using GenomeStudio (Illumina, San Diego, USA) and converted to PLINK format files. The HARVEST project genotyped 33,538 individuals, divided in two batches[18]. The first batch comprising 20,664 individuals and 542,585 SNPs was genotyped at NTNU Genomics Core Facility (Trondheim, Norway) using the Illumina HumaneCoreExome (Illumina, San Diego, USA) genotyping array, version 12 1.1. The second batch, comprising 12,874 individuals and 547,644 SNPs used the Illumina HumanCoreExome genotyping array, version24 1.0. The SELECTIONpreDISPOSED project genotyped 26,990 individuals, in two batches. One batch comprising 17,949 individuals and 692,367 SNPs, and the other batch comprising 9,041 individuals and 692,388 SNPS, both genotyped at ERASMUS MC (the Netherlands) using the Illumina Global Screening Array MD version 1.0 (Illumina, San Diego, USA). The NORMENT project genotyped 174,884 individuals in 21 batches at deCODE genetics (Reykjavik, Iceland). Three batches comprising 25,563 individuals (specifically, the three batches comprised 9,632, 2,426, and 13,505 individuals) and 693,143 SNPs, were genotyped using the Illumina Global Screening Array MD v.1.0. Fourteen batches comprising 120,344 individuals (specifically, the 14 batches comprised 4,418, 24,999, 24,980, 24,995, 4,699, 4,792, 5,625, 4,605, 5,256, 5,446, 2,702, 5,637, 1,971, and 219 individuals) and 687,316 SNPS were genotyped using the Illumina Global Screening Array MD v.3.0. Two batches, comprising 5,959 individuals, were genotyped using the Illumina HumanOmniExpress-24v1.0; one batch comprised 2,976 individuals and 708,882 SNPs, while the other batch comprised 2,983 individuals and 710,146 SNPs. Finally, two batches, comprising 23,018 individuals, were genotyped using the Illumina InfiniumOmniExpress-24v1.2; one batch comprised 17,608 individuals and 712,628 SNPs, while the other batch comprised 713,599 SNPs.

Quality control (QC) was carried out in PLINK version 1.90 beta 3.36 (https://zzz.bwh.harvard.edu/plink/) and KING 2.2.4 based on the Picopili pipeline for family-based data and best-practice QC protocols in human genetics. Pre-imputation QC exclusion criteria for SNPs was used to conduct quality control. Known problematic SNPs previously reported by the Cohorts for Heart and Aging Research in Genomic Epidemiology (CHARGE) consortium and Psychiatric Genomics Consortium (PGC) were excluded from each batch. Duplicate samples were removed, and each genotyping batch was split into parents and offspring. Quality control was then conducted by genotyping array in parents and offspring separately.

Individuals were excluded if they had a genotyping call rate below 95% or autosomal heterozygosity greater than four standard deviations from the sample mean. SNPs were excluded if they were ambiguous (A / T and C / G), had a genotyping call rate below 98%, minor allele frequency of less than 1%, or Hardy-Weinberg equilibrium P-value less than 1 × 10-6. Population stratification was assessed, using the HapMap phase 3 release 3 as a reference, by principal component analysis using EIGENSTRAT version 6.1.4. Visual inspection identified a homogenous population of European ethnicity and individuals of non-European ethnicity were removed. Individuals with a genotyping call rate below 98% or autosomal heterozygosity greater than four standard deviations from the sample mean were then removed. A sex check was done by assessing the sex declared in the pedigree with the genetic sex, which was imputed based on the heterozygosity of chromosome X. When sex discrepancies were identified, the individual was flagged. Relatedness was assessed by flagging one individual from each pairwise comparison of identity-by-descent with a pi-hat greater than 0.1.

The parents and offspring datasets were then merged into one dataset per genotyping batch; keeping only the SNPs that passed quality control in both datasets. All individuals passing the genotyping call rate and autosomal heterozygosity measures were included in the merged datasets. Therefore, the merged datasets included individuals previously excluded or flagged as a duplicate,

ethnic outlier, having a sex discrepancy, or high level of relatedness. Concordance checks were then conducted on validated duplicates. Duplicate, tri-allelic and discordant (any discordance between the validated duplicates) SNPs were excluded. Individuals and SNPs with a genotyping call rate below 98% in the merged datasets were excluded. The duplicate sample that was removed before the start of the quality control was then excluded. Mendelian errors identified by the assessment of duos and trios were then recoded to missing. Insertions and deletions were also excluded.

Phasing was conducted using Shapeit 2 release 837 and the duoHMM approach was used to account for the pedigree structure. Imputation was conducted using IMPUTE4 and the publicly available Haplotype reference consortium (HRC) release 1-1 was used as the genetic reference panel. The Sanger Imputation Server was used to perform the imputation with the Positional Burrows-Wheeler Transform (PBWT). The phasing and imputation were conducted separately for each genotyping batch.

Post imputation quality control was performed by initially converting the dosages to best-guess genotypes. Individuals were removed if they had a genotyping call rate less than 99% or were of non-European ethnicity. SNPs with an imputation INFO quality score less than 0.8, genotyping call rate less than 98%, minor allele frequency less than 1%, or a Hardy-Weinberg equilibrium P-value less than 1 × 10-6 were removed. After quality control, a core homogeneous sample of European ethnicity (based on PCA of markers overlapping with available HapMap markers), unrelated (within generation, defined as accumulated identity-by-descent <0.015 and overall identity-by-descent PI_HAT <10%) individuals across all batches and arrays were available for use in analysis (Nchildren = 28,026).

Supplementary Figure 1. Correlation matrix of the dimensional measures

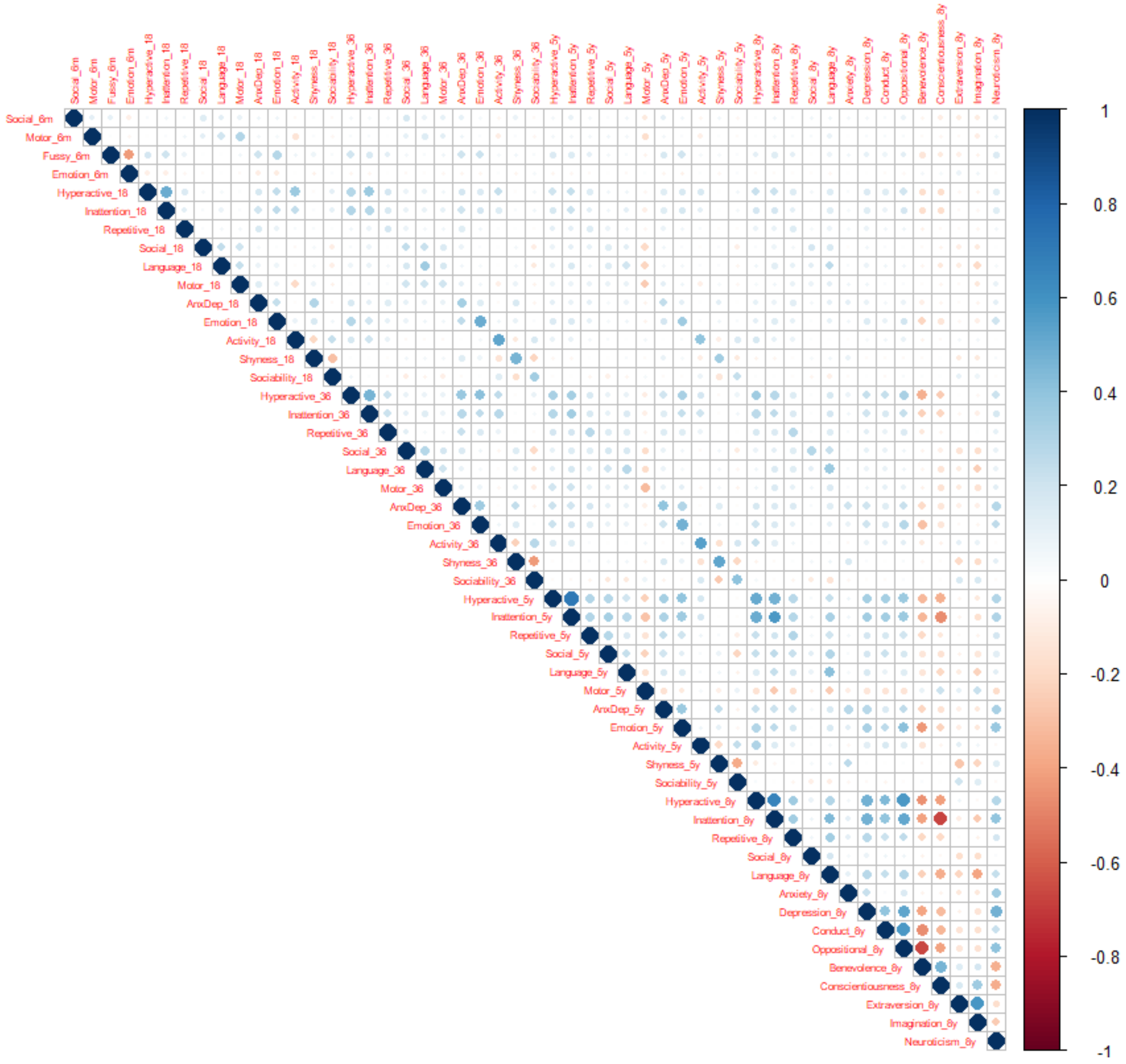

Note: The correlation matrix is in the order by age of the child when the parents answered the questionnaire. Social; social communication difficulties, Motor; motor difficulties, Fussy; fussiness, Hyperactive; hyperactive, Inattention; inattention, Repetitive; repetitive behaviour, Language; language difficulties, AnxDep; Emotional difficulties, Conduct; Conduct difficulties, Oppositional; Oppositional defiant difficulties, 6m; 6 months, 18m; 18 months, 36; 3 years, 5y; 5 years, 8y; 8 years.

Supplementary Figure 2. Equivalence test results with SESOI bounds and widened confidence intervals

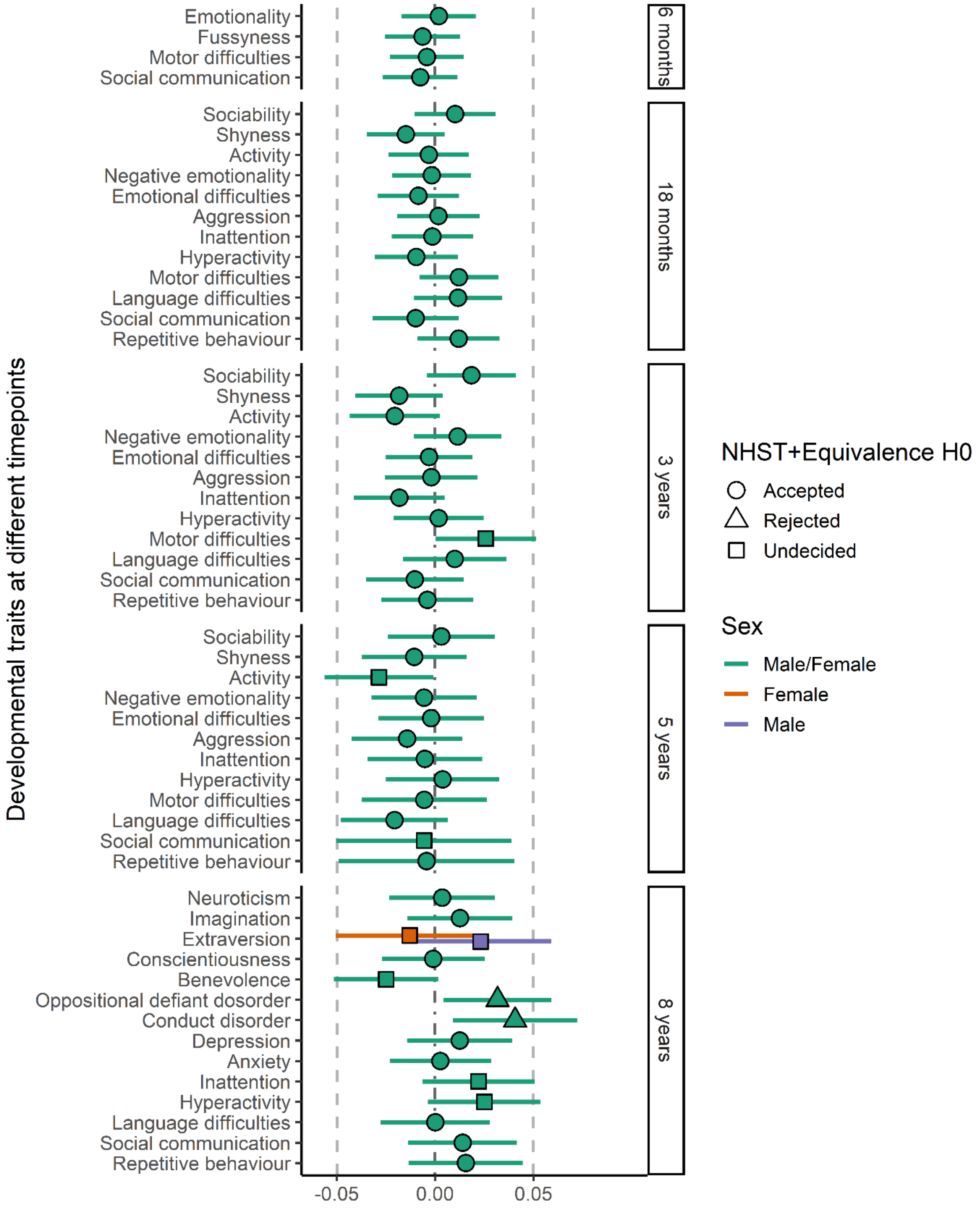

Note: This figure shows the categorizations with the SESOI bounds and widened confidence intervals. Bipolar disorder PRS and developmental outcomes of repetitive behavior, social communication difficulties, language and motor difficulties, hyperactivity, inattention, anxiety, depression, emotional difficulties, fussiness,

emotionality, activity, shyness, sociability, benevolence, conscientiousness, extraversion, imagination, and neuroticism. The null hypothesis in the table refers to a composite null hypothesis of the NHST plus equivalence test. Results presented in a triangle means the composite null test could be rejected. Results presented as circles means they could not be rejected, and results presented as squares means it remains undecided.

Supplementary Figure 3. PRS for Bipolar disorder and association with DSM-5 criteria for oppositional defiant difficulties and conduct difficulties

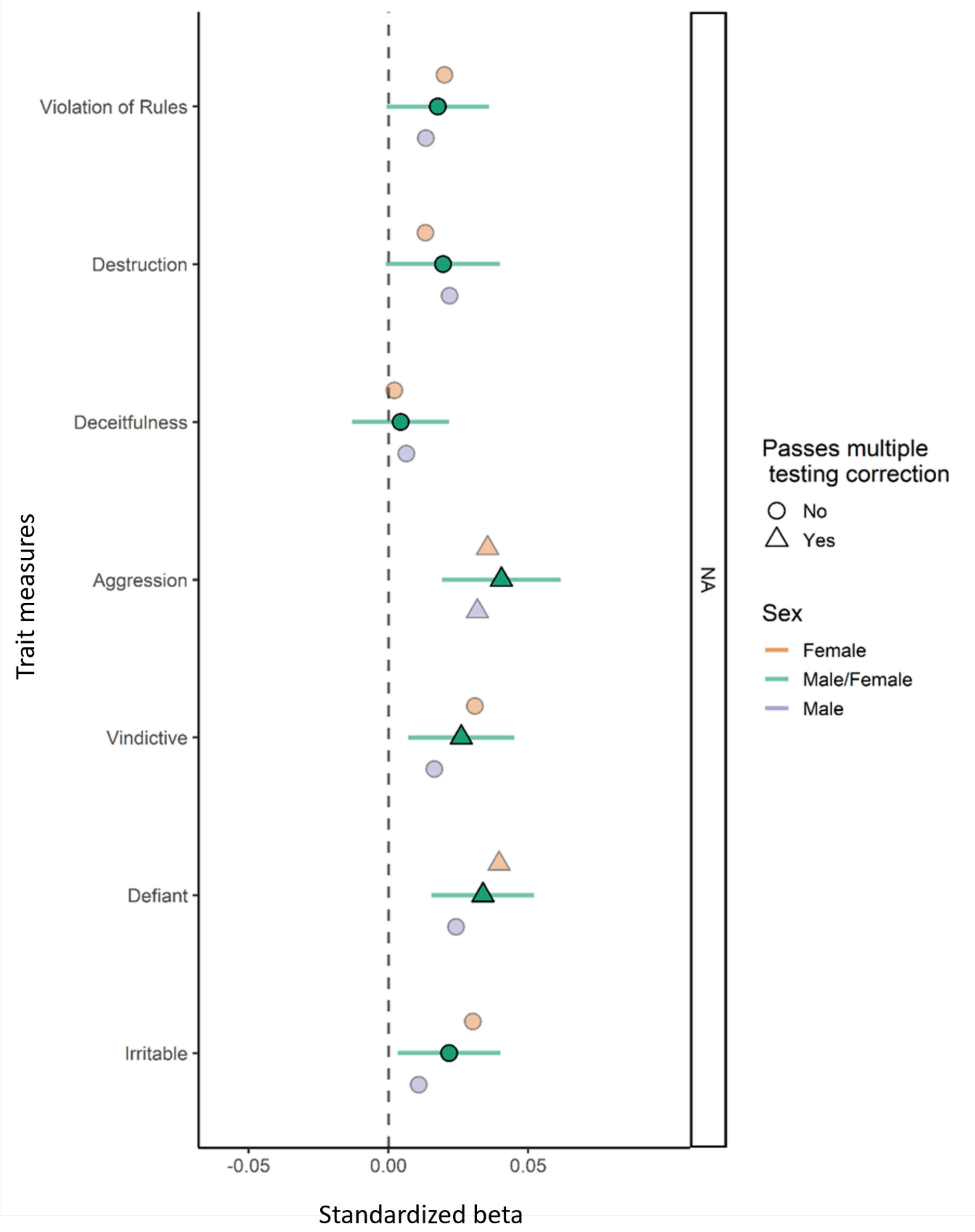

Note: The criteria listed in DSM-5 for conduct difficulties are violation of rules, destruction, deceitfulness, and aggression. The criteria listed in DSM-5 for oppositional defiant difficulties are vindictive, defiant, and irritable mood.

Supplementary Table 1. Equivalence test results for diagnostic measures

Outcome	Estimate	Std. Error	Adjusted 90% LCI	Adjusted 90% UCI	H0
ADHD without conduct disorder	1.122	0.046	1.009	1.249	Undecided
Disruptive behaviors	1.139	0.107	0.887	1.461	Undecided
Autism	1.212	0.125	0.907	1.621	Undecided
Affective disorders	1.183	0.106	0.925	1.514	Undecided
Anxiety disorders	1.079	0.059	0.942	1.237	Undecided

Note: ADHD without conduct disorder (ICD-10 code F900, F908 and F909, n=1738), Disruptive behavior disorders (ICD-10 codes F91, F901 and F92, n=348), Autism spectrum diagnosis (ICD-10 code F84, n=332), Affective disorders (ICD-10 codes F31-F39, n=164) and Anxiety disorders (ICD-10 codes F40, F41 and F93, n=649). Std. Error = standard error, LCI = lower confidence interval, UCI = upper confidence interval.

Supplementary references

1. Squires J, Potter L, Bricker DD. *The ASQ user's guide: a parent-completed, child-monitoring system*. Paul H. Brookes1999.

2. Richter J, Janson H. A validation study of the Norwegian version of the Ages and Stages Questionnaires. *Acta paediatrica* 2007; **96:** 748-752.

3. Robins DL, Fein D, Barton ML, Green JA. The Modified Checklist for Autism in Toddlers: an initial study investigating the early detection of autism and pervasive developmental disorders. *Journal of autism developmental disorders* 2001; **31**(2)**:** 131-144.

4. Rutter M, Bailey A, Lord C. The Social Communication Questionnaire. *Torrance, CA: Western Psychological Services* 2003.

5. Scott FJ, Baron-Cohen S, Bolton P, Brayne C. The CAST (Childhood Asperger Syndrome Test) Preliminary development of a UK screen for mainstream primary-school-age children. *Autism* 2002; **6**(1)**:** 9-31.

6. Bishop DV. *The children's communication checklist: CCC-2*. Harcourt Assessment2003.

7. Achenbach T. Manual for the child behavior checklist/2–3 and 1992. *Department of Psychiatry, University of Vermont, Burlington, VT* 1992.

8. Conners CK, Sitarenios G, Parker JD, Epstein JN. The revised Conners' Parent Rating Scale (CPRS-R): factor structure, reliability, and criterion validity. *Journal of abnormal child psychology* 1998; **26**(4)**:** 257-268.

9. Silva RR, Alpert M, Pouget E, Silva V, Trosper S, Reyes K *et al.* A Rating Scale for Disruptive Behavior Disorders, Based on the DSM-IV Item Pool. *Psychiatric Quarterly* 2005; **76**(4)**:** 327-339.

10. Ireton H. Child development inventory. Behavior Science Systems Minneapolis, MN1992.

11. Birmaher B, Khetarpal S, Brent D, Cully M, Balach L, Kaufman J *et al.* The screen for child anxiety related emotional disorders (SCARED): Scale construction and psychometric characteristics. *Journal of the American Academy of Child & Adolescent Psychiatry* 1997; **36**(4)**:** 545-553.

12. Birmaher B, Brent DA, Chiappetta L, Bridge J, Monga S, Baugher M. Psychometric properties of the Screen for Child Anxiety Related Emotional Disorders (SCARED): a replication study. *Journal of the American academy of child & adolescent psychiatry* 1999; **38**(10)**:** 1230-1236.

13. Angold A, Costello EJ. Mood and feelings questionnaire (MFQ). *Durham, NC: Developmental Epidemiology Program, Duke University* 1987.

14. Messer SC, Angold A, Costello EJ, Loeber R, Van Kammen W, Stouthamer-Loeber M. Development of a short questionnaire for use in epidemiological studies of depression in children and adolescents: Factor composition and structure across development. *International journal of methods in psychiatric research* 1995; **5:** 251-262.

15. Bates JE, Freeland CAB, Lounsbury ML. Measurement of infant difficultness. *Child development* 1979**:** 794-803.

16. Buss AH, Plomin R. *Temperament: Early developing personality traits*. Hillsdale, NJ: Lawrence Erlbaum Associates1984.

17. Construction of the Hierarchical Personality Inventory for Children (HiPIC). *Proceedings of the Personality psychology in Europe. Proceedings of the Eight European Conference on Personality Psychology/I. Mervielde, I. Deary, F. De Fruyt, & F. Ostendorf (Eds.).-Tilburg: Tilburg University Press, 1999*1999.

18. Helgeland Ø, Vaudel M, Juliusson PB, Holmen OL, Juodakis J, Bacelis J *et al.* Genome-wide association study reveals dynamic role of genetic variation in infant and early childhood growth. *Nature communications* 2019; **10**(1)**:** 1-10.
